# Comparison of sputum microbiome of legionellosis-associated patients and other pneumonia patients: indications for polybacterial infections

**DOI:** 10.1038/srep40114

**Published:** 2017-01-06

**Authors:** Hila Mizrahi, Avi Peretz, René Lesnik, Yana Aizenberg-Gershtein, Sara Rodríguez-Martínez, Yehonatan Sharaby, Nina Pastukh, Ingrid Brettar, Manfred G. Höfle, Malka Halpern

**Affiliations:** 1 Department of Evolutionary and Environmental Biology, Faculty of Natural Sciences, University of Haifa, Mount Carmel, Haifa, Israel; 2 Microbiology Lab, Baruch Padeh Medical Center, Poriya, affiliated with the Faculty of Medicine, Bar Ilan University, Israel; 3 Department of Vaccinology and Applied Microbiology, Helmholtz Centre for Infection Research (HZI), Braunschweig, Germany; 4 Department of Biology and Environment, Faculty of Natural Sciences, University of Haifa, Oranim, Tivon, Israel

## Abstract

Bacteria of the genus *Legionella* cause water-based infections resulting in severe pneumonia. Here we analyze and compare the bacterial microbiome of sputum samples from pneumonia patients in relation to the presence and abundance of the genus *Legionella*. The prevalence of *Legionella* species was determined by culture, PCR, and Next Generation Sequencing (NGS). Nine sputum samples out of the 133 analyzed were PCR-positive using *Legionella* genus-specific primers. Only one sample was positive by culture. Illumina MiSeq 16S rRNA gene sequencing analyses of *Legionella-*positive and *Legionella-*negative sputum samples, confirmed that indeed, *Legionella* was present in the PCR-positive sputum samples. This approach allowed the identification of the sputum microbiome at the genus level, and for *Legionella* genus at the species and sub-species level. 42% of the sputum samples were dominated by *Streptococcus. Legionella* was never the dominating genus and was always accompanied by other respiratory pathogens. Interestingly, sputum samples that were *Legionella* positive were inhabited by aquatic bacteria that have been observed in an association with amoeba, indicating that amoeba might have transferred *Legionella* from the drinking water together with its microbiome. This is the first study that demonstrates the sputum major bacterial commensals and pathogens profiles with regard to *Legionella* presence.

*Legionella* is a genus of aquatic Gram-negative, rod-shaped, facultative aerobic bacteria. Members of the genus *Legionella* are found around the globe in a variety of natural and man-made freshwater environments^1^,^2^. Protozoa are considered the natural hosts of *Legionella* in these environments^3^. *Legionella* spp. and especially *L. pneumophila*, the clinically most relevant species, can cause two clinical syndromes in humans. The first is Pontiac fever, a self-limited flu-like illness that develops within 2–3 days in 95% of the people that are exposed to the bacteria. The second form is legionellosis or Legionnaires’ disease (LD), a severe form of pneumonia that could also affect other organs, such as the liver and kidneys^4^,^5^. Humans become infected with *L. pneumophila* by inhaling aerosols from aquatic environments, such as potable water, cooling towers, showerheads, whirlpools, and other man-made devices that generate aerosols^6^. LD is not transferred from human to human, i.e. it is not a communicable disease^2^,^7^. Major risk factors for developing the disease include suppression of the cellular immune system, cigarette smoking, use of well water, and chronic heart or lung disease^8^. The conventional antibiotic treatment includes azithromycin, fluoroquinolones (levofloxacin, moxifloxacin), and sulfamethoxazole - trimethoprim^9^,^10^. Despite antibiotic treatment, the case fatality rate for LD in hospitalized patients remains high, with reported rates of 5–30%^11^.

The epidemiological features of legionellosis in Israel are rather similar to those in the EU regarding incidence rates, seasonality, and the methods used for laboratory diagnosis. However, in Israel a larger proportion of nosocomial cases occurred^12^. An average of 49.3 LD cases was diagnosed annually between 2006 and 2011 in Israel; 71.4% of the clinical isolates belonged to *L. pneumophila* serogroup 1^12^. Indeed, *L. pneumophila* serogroup 1 was recently reported as the dominant serogroup isolated from the drinking water system in Israel^13^,^14^. It is difficult to clinically distinguish LD from other causes of pneumonia; thus, many legionellosis cases remain unreported. It is highly important to develop a clinical scoring system to distinguish LD from other pneumonia causes^15^. Today there are several diagnostic tests in use for the detection of *Legionella* infections such as: Culture diagnosis, Urinary Antigen Detection, Direct Fluorescent Antibody (DFA) staining, and *Legionella-*specific PCR^8^. Previously, culture diagnosis was the golden standard for legionellosis diagnosis. Nowadays it is hardly used in routine diagnosis because it is unable to provide results within a clinically useful time frame as the diagnosis takes between three to five days^16^. The DFA staining is commonly used however, this test lacks sensitivity for detecting all clinically important *Legionella* species^17^. Thus, LD can be described as an elusive diagnosis rather than an exotic infection^16^. Although molecular tools such as specific PCR for *Legionella* spp. PCR have been developed, they are rarely used in clinics^18^. In Europe, only 2% of the 11,832 confirmed or probable LD cases were ascertained by PCR^19^.

In recent years, Next Generation Sequencing (NGS) high-throughput technologies have been applied to study the human microbiome–the bacterial communities inhabiting humans^20^. Using these methods, the microbiota of different human body organs can be studied directly using DNA extracted from samples and without employing any culture techniques. Currently, several studies investigated the lungs, the respiratory tract, and the sputum microbiome^21^,^22^,^23^,^24^ with regard to tuberculosis or cystic fibrosis. Nevertheless, none of them studied the sputum microbiome with regard to legionellosis. A better understanding of the microbiota associated with pneumonia patients may improve our understanding of patients with pulmonary infections and particularly patients with LD.

The aim of the current study was to analyze the bacterial community and the proportion of *Legionella* in sputum samples of patients with pneumonia due to *Legionella* spp. and to compare it to sputum samples of patients with pneumonia due to other pathogens. Using a NGS approach based on 16S rRNA gene amplicons, we compared bacterial communities of sputum of pneumonia patients with respect to richness, diversity, and relative abundances of bacterial genera in correspondence with presence and abundance of the genus *Legionella* and *L. pneumophila.*

## Results

### Screening of sputum samples for *Legionella*

One hundred and thirty-three sputum samples were analyzed for the presence of *Legionella* species by both culture and molecular methods. To verify the ability of the chosen molecular method to detect the presence of *L. pneumophila* in a sputum sample, the sensitivity of the PCR was tested as follows: sputum samples were inoculated with known concentrations of *L. pneumophila* and DNA was extracted from the inoculated samples. The method was proven to be very sensitive as *Legionella* were still detectable in sputum samples that were inoculated with only 40 cfu/ml using the *Legionella* genus-specific PCR reaction (unpublished data). Nine sputum samples (6.8%) were positive for *Legionella* by PCR. One sample (sample 2PS2) out of these nine PCR-positive samples was also positive by culture. The isolate was identified as *L. pneumophila* serogroup 1. Details regarding the patients are summarized in Table S1.

### NGS analyses of the sputum microbiome

Bacterial communities of the nine *Legionella-*positive sputum samples (LGP) and 13 *Legionella-*negative samples (LGN, chosen randomly) were studied using Illumina MiSeq deep sequencing. One of the LGP samples had to be discarded due to insufficient number of sequences. Across all 21 sputum samples analyzed, almost 2 million quality sequences were obtained. Those sequences were classified in total as 64,965 unique OTUs (Operational Taxonomic Units) at the 97% sequence similarity level across all samples. Sequences were classified into 12,738 OTUs after rarefying of all the samples to the lowest number of reads (7,155 sequences). At 3% sequence divergence, most rarefaction curves describing the number of OTUs observed as a function of sampling effort asymptotically approached saturation, indicating that the surveying effort covered almost the full extent of taxonomic diversity at this phylogenetic level (Fig. S1). Detailed coverage and Chao1values of the OTUs are given in Table S2. A Principal Coordinate Analysis (PCoA) was conducted in order to have a global picture on the relations between LGP and LGN samples. The AMOVA that was used to assess the statistical significance of the separation among the LGP and LGN groups did not show significant dissimilarities (F_1,19_ = 1.11, *p* = 0.28).

### Taxonomic composition of the sputum samples

The sputum samples were analyzed by *Legionella*-specific PCR and bacterial-specific NGS. Based on the PCR results the samples were qualified as *Legionella* positive (LGP) and *Legionella* negative (LGN). The PCR results were comparable to the NGS results, namely, *Legionella* sequences were detected in the NGS in all of the *Legionella* PCR-positive samples. For further analyses, we compared the three samples with *Legionella* abundance above 0.5% separately (samples 2PS2, 6PS2, and 26PS4 with 2.88%, 0.82%, and 0.56% *Legionella* abundance, respectively), ‘High-LGP’ hereafter. The other five *Legionella* PCR-positive samples had a far lower abundance ranging from 0.11% to 0.02% and sample 33PS3 with only 0.004%. These samples are referred to as ‘Low-LGP’ hereafter.

At the phylum level, *Legionella* positive (LGP) and negative (LGN) samples had a comparable composition with a pronounced dominance of *Proteobacteria* and *Firmicutes* that together account for more than 80% of the bacterial community (Fig. S2A). Together with *Actinobacteria* (up to 9%) and *Bacteroidetes* (up to 5%) these four phyla comprised more than 96% of the total bacterial community. A very distinct pattern was observed in the high and low *Legionella* samples. *Proteobacteria* (High-LGP 66%, Low-LGP 27%) and *Actinobacteria* (High-LGP 11%, Low-LGP 4%) had an almost three-fold higher abundance in high *Legionella* samples (Fig. S2B). *Firmicutes* was the dominant phylum for low *Legionella* abundant samples (High-LGP 16%, Low-LGP 63%) (Fig. S2B). The higher abundance of *Firmicutes* is mainly due to the high abundance of *Streptococcus*.

**Figure 1:**
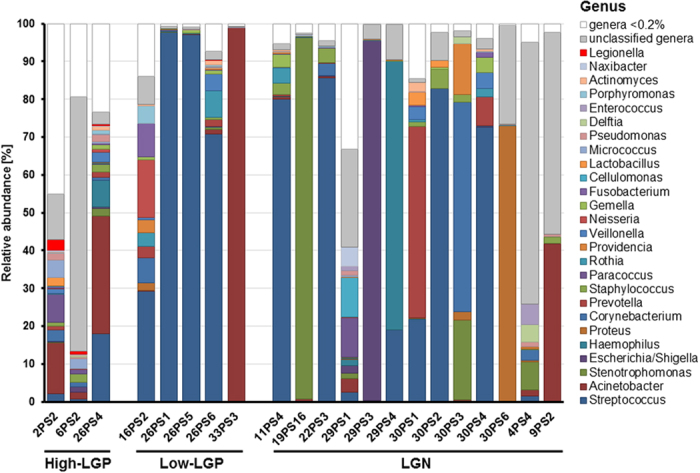
Microbiome composition of all sputum samples analyzed by NGS at genus level. Classified genera with relative abundances above a cut-off level of 0.2% are indicated. White bars comprise of all the genera with a relative abundance of <0.2%. Gray bars represent taxa with a relative abundance above the cut-off level of 0.2%, but that could not be classified at genus level. High *Legionella* load (High-LGP), low *Legionella* load (Low-LGP) and *Legionella* negative samples (LGN) are displayed as separate groups for comparison.

The genus composition of each sample is shown in Fig. 1. At the genus level, the *Legionella*-positive sample types show both a comparable high abundance of *Acinetobacter* and a highly distinct abundance of *Streptococcus* that constitutes a major fraction of the Low-LGP samples. For the *Legionella* negative samples (LGN), six more genera achieved higher abundances (*Stenotrophomonas, Escherichia-Shigella complex, Haemophilus, Proteus, Corynebacterium,* and *Prevotella*) (Fig. 1). The High-LGP group can be distinguished from the Low-LGP and the LGN on the genus level. A main distinction for the High-LGP is a reduced dominance of single genera, i.e., only in one case does a single genus exceed an abundance of 18%, and a broad set of different genera ranges between 2% and 0.2% abundance. By contrast, the Low-LGP group is usually dominated by a single genus, mostly *Streptococcus*. The LGN samples are mostly (10 out of a total of 13 samples) dominated by a single genus; however, there is a broad set of different genera that may dominate these sputum samples. The genus *Streptococcus* played a dominant role for four samples. Seven LGN samples were each dominated by a different genus (*Acinetobacter, Stenotrophomonas, Escherichia-Shigella-complex, Haemophilus, Proteus, Corynebacterium,* and *Prevotella*). The remaining two LGN samples did not have dominant genera, mostly due to the presence of a high fraction of non-classified genera (Fig. 1).

### Genus diversity and genera origin in sputum samples

**Figure 2:**
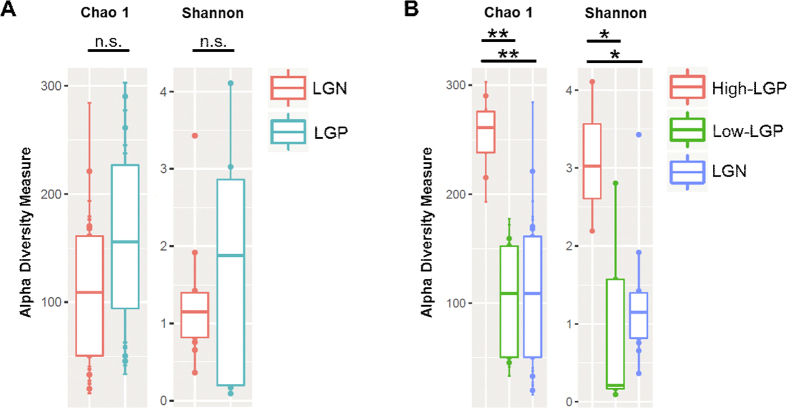
Alpha-Diversity estimates for sputum samples based on OTU-data at the genus level. **(A**) Comparison of *Legionella*-negative samples (LGN) with the total of the *Legionella*-positive samples (LGP) was not distinct. (**B**) Samples with high abundance of *Legionella* (High-LGP) display a higher diversity on the genus level than samples with low *Legionella* abundance (Low-LGP) and PCR-negative samples (LGN). Levels of significance are indicated by asterix as inferred from pairwise t-test with Holms-adjusted p-values (**p-value ≤ 0.01, *p-value ≤ 0.05, n.s.: not significant; more details can be found in Table 1). This pattern was also observed for these samples on the family level. For a comparison of different diversity indices on the genus and family levels see also Supporting Information (Fig. S3) and Table 1.

Chao1 and Shannon indices were calculated at the genus level for *Legionella* positive and negative samples, and in addition, High-LGP (0.56% to 2.88% relative abundance) and Low-LGP (0.11% to 0.01% relative abundance) (Fig. 2). While richness and diversity were not distinct when comparing *Legionella*-positive vs. *Legionella-*negative samples, there was a clear distinction of High-LGP from Low-LGP or LGN samples. Richness and diversity were significantly higher for High-LGP compared to Low-LPG and LGN (p < 0.05, Fig. 2 and Table 1). This pattern was also observed using other diversity indices for the genus and the family level (Fig. S3).

**Figure 3:**
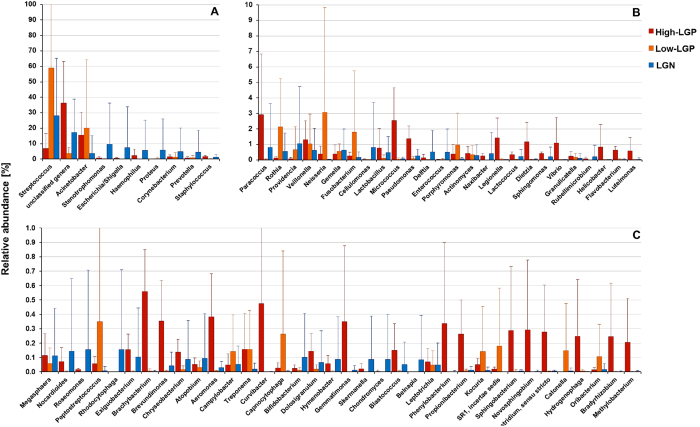
Rank abundance curves of the overall genus composition of the sputum microbiomes at different levels of abundances. Shown are the average relative abundances for all genera that exceeded 0.5% relative abundance in at least one sample. Samples are grouped by relative *Legionella* abundance, i.e. high *Legionella* load (High-LGP; red, i.e. >0.5% relative abundance), low *Legionella* load (Low-LGP; orange, i.e. 0.11 to 0.01% relative abundance) and where *Legionella* was not detectable by PCR (LGN; blue). For comparison of genera at different abundance levels, three separate plots showed genera in the range of 1–100% (**A**), 0.1–10% (**B**) and 0.01–1% (**C**) relative abundance. For detailed abundance values, see also Supporting Information (Tables S3 and S4).

Details on the genus composition of the sputum samples are shown in Fig. 3 and in Tables S3 and S4. The average of the genera abundances is presented at three different abundance levels (Fig. 3A, from 1.0% to 100%; Fig. 3B from 0.1% to 10%; and Fig. 3C from 0.01% to 1%). High-LGP samples had different genera than Low-LGP or *Legionella* negative (LGN) samples. The High-LGP samples showed high genus diversity between 2% and 0.2% relative abundance. Though still distinct, the genus composition within the High-LPG samples was more comparable than those within the Low-LGP and the LGN samples (Fig. 3).

An interesting observation was that in most of the sputum samples, OTUs belonging to one or two genera were dominant. For example, *Streptococcus* OTU abundance in samples 26PS1, 26PS6, 11PS4, 22PS3, 30PS2 and 30PS4, were 97.5%, 70.9%, 80.1%, 85.7%, 82.8% and 72.7%, respectively. *Acinetobacter* OTUs abundances in samples 33PS3 and 9PS2 were 98.9% and 41.5%, respectively. *Stenotrophomonas* OTUs abundances in sample 19SP16 were 95.7%. (Tables S3 and S4). Sample 16PS2 was dominated by *Streptococcus* (29.2%) and *Neisseria* (15.2%); sample 26PS4 was dominated by *Streptococcus* (17.9%) and *Acinetobacter* (31.3%); sample 30PS1 was dominated by *Prevotella* (50.6%) and *Streptococcus* (21.8%); and sample 30PS3 was dominated by *Corynebacterium* (55.6%) and *Stenotrophomonas* (21.0%) (Tables S3 and S4)

### NGS results compared to hospital laboratory culture results

Sputum samples are cultured by the hospital laboratory on selective and diagnostic plates only if they have more than 25 leukocytes per microscopic field. This is why, seven out of the 21 Illumina analyzed sputum samples were not cultured at all. NGS analyses showed the prevalent genus of the sputum samples (Tables S3 and S4), however, in the majority of cases, these results did not match the hospital laboratory culturable results. The culturable results of only two samples (2PS2 and 29PS3) matched the NGS result, i.e., *Legionella* was detected on a selective medium, and *Escherichia* was identified as the most abundant pathogen. In all other cases, abundant species were not detected; in some cases bacteria of minor prevalence in the NGS analysis were cultured. Yet, it has to be emphasized that commensal pathogens (for example streptococci which can be normal oral microbiota), are not reported by the clinical laboratory, while NGS sequencing is unable to resolve pathogenic streptococci species.

### Comparative sequence analysis of *Legionella* species

**Figure 4:**
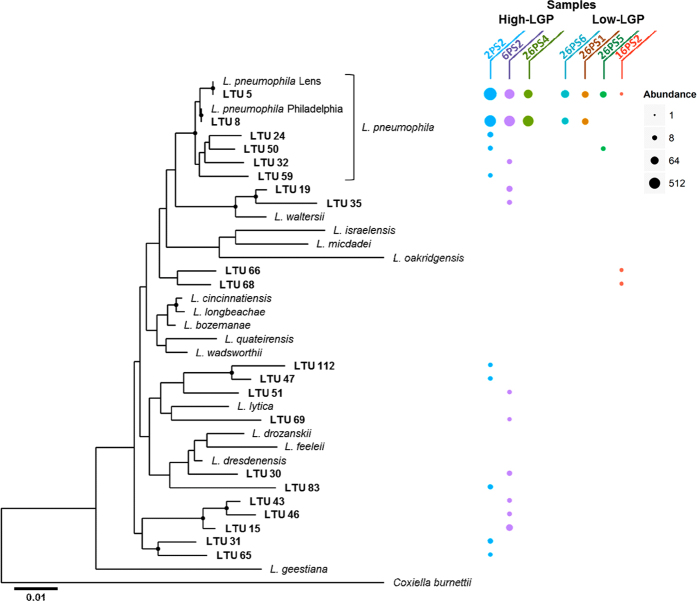
Phylogenetic diversity and abundance of *Legionella* operational taxonomic units (LTUs) in *Legionella*-positive (LGP) sputum samples based on 16S rRNA sequence comparison. Representative sequences of each LTU were compared with selected reference sequences of described *Legionella* species. *Coxiella burnettil* served as an outgroup. The size of the colored dots represents LTU-specific abundances in read numbers. The colors refer to the different samples. LTUs display a sample-specific distribution pattern and those belonging to the *L. pneumophila* branch (bracket) were most abundant. For clarity, only clusters with ≥5 reads are shown. Sample 33PS3 was below this threshold. Nodes with bootstrap support of ≥50% are indicated and the scale bar shows the number of substitutions per site.

**Figure 5:**
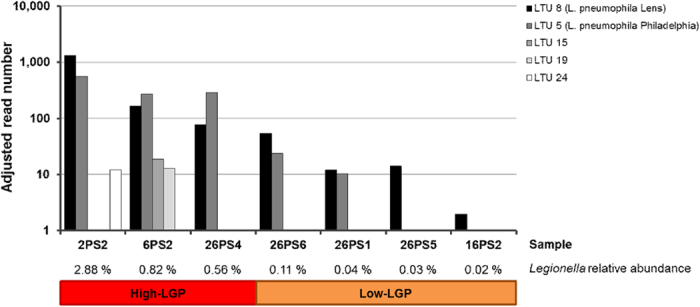
Distribution of the most abundant *Legionella* operational taxonomic units (LTUs) in *Legionella*-positive (LGP) sputum samples with high (red) and low (orange) *Legionella* load. For clarity only clusters with more than 10 reads in at least one sample are shown. Sample 33PS3 was below this threshold. Note that the scale is log-transformed.

The 16S rRNA gene amplicons analyzed in this study allowed differentiation between most *Legionella* species and also between two clusters of *L. pneumophila* at the subspecies level: One cluster: *Legionella* operational taxonomic unit 8 (LTU 8) comprised *L. pneumophila* reference strain Philadelphia (and included also Corby and Fraseri). A second cluster (LTU 5) comprised *L. pneumophila* Lens (and Alcoy) (Fig. 4). Clustering at the 99% sequence similarity level showed that the majority of *Legionella* reads in LGP sputum samples were indeed clustering with one of the two aforementioned *L. pneumophila* clusters (Table S5 and Fig. 4). Phylogenetic comparison further elucidated the prevalence of sequences highly similar to *L. pneumophila* and the sample-specific compositions of LTUs (Fig. 4). Only in sample 16PS2 non-pneumophila LTUs (LTU 66 and 68) were slightly more abundant than LTU 5 (Fig. 4). Interestingly, non-pneumophila LTUs displayed an even more sample-specific distribution pattern by each cluster, occurring only in a single sputum sample (2PS2 and 6PS2). The fact that the abundance pattern of LTUs differed substantially between all samples indicated that sequence variations were not due to amplification or sequencing errors but might reflect the individual nature of each *Legionella* infection. By looking at the phylogenetic distribution of the major LTUs detected in the sputum samples it is apparent that, in addition to the two subspecies represented by the strains Philadelphia and Lens, a third phylogenetic branch exists that was detected in three of the patients (26PS5, 2PS2, and 6PS2) comprising the LTUs 24, 50, 32, and 59 from *L. pneumophila* (Fig. 4). Therefore, we conclude that there might be a third *L. pneumophila* subspecies specifically occurring in the respective patients in Israel. In addition, the two samples (2PS2 and 6PS2) with the highest relative abundance of *L. pneumophila* reads, had sets of 5 and 8 and non-pneumophila LTUs associated with the *L. pneumophila-*specific LTUs (Fig. 4). This hints at additional signatures for a patient-specific NGS profile of *Legionella* species that could be of clinical relevance. The distribution of the most abundant LTUs in High-LGP and Low-LPG sputum samples is presented in Fig. 5.

## Discussion

LD or Legionellosis is a type of bacterial pneumonia caused by *L. pneumophila* and other pathogenic *Legionella* species^8^. Studies in Europe and the United States showed that *Legionella* infections are responsible for 1–5% of all hospitalized community-acquired pneumonia cases^24^. LD identification in pneumonia cases is underestimated due to the failure to diagnose LD in routine practice^15^. Several attempts at developing a clinical scoring system to distinguish LD from other pneumonias have failed^8^. In the current study we monitored the prevalence of *Legionella* in hospitalized pneumonia patients by both culture-dependent and -independent (PCR) methods. In addition, we used the Illumina MiSeq platform to sequence the 16S rRNA gene in order to describe the relation between *Legionella* and the composition of the sputum microbiome. *Legionella* detection by culture-dependent methods is limited compared to the PCR-based culture-independent methods, in which indicative genes are assessed directly from the DNA extracted from sputum^25^.

The prevalence of *Legionella* species in sputum samples by PCR genus-specific primers according to Kahlisch *et al*.^26^ was 6.8% with nine out of 133 positive samples. The PCR reaction was found to be very sensitive, as it could detect the presence of 40 *Legionella* CFUs per ml. In contrast, *Legionella* was cultured only from one sample (2PS2). Patient 2PS2 was the only one recognized by the hospital as having LD. As for the other eight patients that were *Legionella*-positive only by PCR, there was a debate regarding their treatment. However, they received an antibiotic treatment that also covered LD. There is no reference in the literature discussing similar cases, and bacterial inoculum required to cause human infection or disease is currently under debate^8^,^27^,^28^,^29^.

The NGS analyses of the sputum microbiome revealed that a broad set of bacteria were dominating the sputum, especially for samples without higher abundance of *Legionella* (LGN and Low-LGP). *Streptococcus* played a major role in these samples. However, in LGN samples six more genera showed high abundances, i.e., *Stenotrophomonas, Escherichia-Shigella complex, Haemophilus, Proteus, Corynebacterium,* and *Prevotella* (Fig. 3, Tables S3 and S4). Besides the highly abundant genera, a second or a third bacterial genus with a higher abundance (above 10% relative abundance) was observed. Interestingly, *Legionella* was never a dominant genus and ranged below 2.9% of relative abundance, even in the case of confirmed *Legionellosis*.

The bacteria in sputum with high *Legionella* abundance (>0.5% relative abundance) showed a distinct composition and diversity compared to samples without or with low *Legionella* abundance. This was indicated by a significantly higher richness and diversity of the high *Legionella* samples (p < 0.05, Fig. 2 and Table 1). Furthermore, the composition on the phylum and the genus level was distinct; especially pronounced was the lower abundance of *Firmicutes* in the high-LGP samples.

The genus composition of High-LGP samples was distinct in many respects from the LGN and Low-LGP samples (Figs 1 and 3). High-LGP samples showed a lower pronounced dominance of single genera and high genus diversity between 0.2% and 2% relative abundance. A large fraction of genera of the High-LPG can be considered of environmental origin, with many of aquatic origin. In addition to *Legionella*, waterborne pathogens such as *Acinetobacter, Stenotrophomonas, Pseudomonas, Vibrio, Helicobacter,* and *Aeromonas* were present. Some genera that were found in the High-LGP samples were observed in the amoeba microbiome isolated from drinking water distribution systems^30^,^31^,^32^. These include protobacterial genera of aquatic origin such as the *Alphaproteobacteria* genera *Sphingomonas, Brevundimonas, Novosphingobium, Bradyrhizobium* and *Methylobacterium*, the *Betaproteobacteria* genus *Curvibacter*, and the *Gammaproteobacteria* genera *Legionella, Acinetobacter, Stenotrophomonas, Escherichia, Pseudomonas,* and *Sphingobacterium.* These potentially amoeba-borne genera form a fraction between 24.4% and 5.6% of the total genera (including unassigned and unclassified) or 8% to 33% of the classified genera. Thomas *et al*.^32^ highlighted the potential risk associated with these amoeba-based bacteria from drinking water systems. The fraction of genera of “potential amoebal origin” increased with higher *Legionella* abundance. For a comparison, the genus *Legionella* itself contributed 9% to 4% of this “potentially amoeba-borne fraction”. In contrast, in LGN samples, only three *Gammaproteobacteria* genera with shown presence in amoeba in drinking water were occasionally observed, i.e., *Acinetobacter, Stenotrophomonas,* and the *Escherichia-Shigella*-complex, whereas the other genera were not detected or were much less abundant (Fig. 3).

*L. pneumophila* was the most prevalent *Legionella* species in LGP samples. The High-LPG samples had not only the highest *Legionella* abundance, but also the highest sub-species *L. pneumophila* diversity and non-pneumophila phylotype diversity compared to the Low-LGP samples (Fig. 4). Based on the above observations, we hypothesize that the presence of high *L. pneumophila* abundance in our samples might have been caused by the transfer of amoeba or amoebal vesicles together with the co-microbiome of the amoeba. This might explain the establishment of *L. pneumophila* due to their greater virulence after contact with amoeba and the accompanying microbiota that is distinct from the other sputum samples.

*Streptococcus* was a prevalent genus in almost half of the LGN samples and 80% of the Low-LGP samples (Tables S4 and S5). These results are in agreement with Cho *et al*.^33^ who described *Streptococcus pneumoniae* as the most common bacterial agent in community-acquired pneumonia. *Streptococcus* was also found to be the most abundant genus in the lung microbiomes of cystic fibrosis patients^23^,^33^. Besides *Streptococcus*, there were seven more pathogenic genera dominating LGN samples (Fig. 3A and Table S5). Although *Streptococcus* may have an outstanding role, a broad set of other genera was shown to dominate sputa and can be considered as causes of pneumonia^29^,^34^.

For LGP samples, at least one more dominant bacterial species was present suggesting that coinfection of *Legionella* with another species may occur (Fig. 1 and Table S3). An interesting point that arises here is that *Legionella* was never the dominating genus, and was always accompanied by other respiratory pathogens. The pattern was distinct for samples with high and low *Legionella* abundance. For low-LGP samples, there was mostly dominance by *Streptococcus*, except for one sample with dominance of *Acinetobacter*. For the high-LGP samples, *Acinetobacter* and *Streptococcus* co-occurred but with a much lower abundance. Therefore, for the High-LGP patients, disease due to *Legionella* is more likely than for the Low-LGP patients. Tan *et al*.^35^, described six LD patients who all had bacteremic co-infection of *Legionella*, particularly with *Streptococcus pneumoniae. Legionella* coinfection cases with other bacterial species were also described in the litrature. For example; coinfections of *Legionella* with *Mycoplasma pneumoniae, Chlamydia pneumoniae, Chlamydia psittaci, Klebsiella pneumoniae* and *Pseudomonas aeruginosa*^36^ and *Listeria monocytogenes*^37^. Coinfections of *Legionella* with the fungus *Pneumocystis jirovecii* in an infant^38^, with influenza virus^39^ and with herpesvirus^40^, have also been reported. Based on our results and the literature evidence, we hypothesize that *Legionella* patients might have bacterial coinfections.

Comparison between NGS and the culture results that were obtained by the hospital laboratory were highly divergent (Table S6). In many cases, species at a low abundance in NGS were cultured. These differences can be due the fact that the bacterial community in a sputum sample is a mixture of different species, and thus a species with a minor prevalence but a more competitive growth in culture overgrew others and is identified as the disease causing agent (for example; *Acinetobacter* can overgrow *Legionella* even on *Legionella*-selective medium). In a former large cohort study of Cystic Fibrosis patients we^23^ have shown that the coincidence between NGS data and clinical cultivation based data may be highly consistent. The full spectrum of bacterial genera can be very helpful to judge the patient’s development and response to the treatment^23^. In the current study, very heterogeneous group of pneumonia patient were analyzed, and as shown, infected by a broad set of different bacterial genera. This may explain the lower match between clinical culture based and NGS data, because it is challenging to cover such a broad spectrum of bacterial genera by cultivation. Charlson *et al*.^41^ and Morris *et al*.^42^ suggested that the upper and lower airway bacterial community composition is rather comparable within healthy individuals, except that the bacterial abundance decreases towards the lung tissue. Moreover, some bacterial species tend to increase in the lung compared to the upper airways^43^. Recently, Segal *et al*.^44^, showed that for healthy volunteers, the lung microbiome can be similar or can differ from the upper airway microbiome. They showed that inflammatory processes occurring in healthy airways lead to a high similarity between the upper airway microbiome and the lung microbiome. They^44^ concluded that the transfer is based on increased microaspiration caused by inflammatory processes. Thus, sputum samples can be considered as providing a good assessment of relevant pathogens of the overall respiratory system. This was also evident from our former study^23^. Our results combined with the evidence from the literature demonstrate that there is a need to reevaluate the regulation of medical laboratory routine procedures and to consider using molecular methods for more accurate diagnosis.

In Figs 4 and 5 *Legionella* species-specific NGS profiles (based on LTUs) provided most patient-specific signatures, i.e., the abundances of the individual LTUs vary from patient to patient. While the microbiological understanding of these profiles awaits further studies at a strain level (also conceivable with marker genes of higher resolution), the highly diverse profile could help identify the source of infection if similar profiles would be obtained from DNA extracted from suspected sources. Coscollá *et al*.^45^, also described mixed infections of *L. pneumophila* strains in outbreak patients. They analyzed sequence based typing (SBT) profiles from uncultured respiratory samples and found evidence of a mixture of SBT *Legionella* profiles in three of the patients. These, combined with our results indicate that patients may be infected from the environment by more than one strain.

The suggested polymicrobial nature of *L. pneumophila* infections bears essential aspects for the survival and growth of *L. pneumophila* on one hand, and the development of the disease for the patient on the other hand. Several studies have shown that *L. pneumophila* is not a very competitive bacterium^46^,^47^. Both positive and negative interactions of *L. pneumophila* with the co-occurring microbiota are highly likely to occur and thus may have an important impact on its proliferation and the resulting disease. The distinct bacterial community occurring in the presence of high *Legionella* abundance may be an indication in this respect.

With respect to the competitiveness of *L. pneumophila* and its interaction with the co-occurring microbiota, the strain level may be of further interest. The diversity of the present *L. pneumophila* strains within the microbiota along the whole respiratory tract may represent an important selection factor. As revealed by the analysis of *Legionella* clusters in this study, the diversity was greater with increasing *Legionella* abundance (Figs 2 and S3). Based on our observations, we hypothesize that a high diversity supported the competitiveness of *L. pneumophila* in the lung microbiota, but this still has to be substantiated by more detailed studies on the strain level. Based on our data, we think that future studies analyzing the interactions between *L. pneumophila* and the co-occurring microbiota (including non-bacterial pathogens), are valuable and needed. A more thorough understanding of these interactions can be considered to hold great promise for the prevention of legionellosis.

### Future perspectives

Overall, our sputum NGS sequencing resulted in profiles of all major bacterial commensals and pathogens for individual patients. We analyzed the microbiota profiles at different taxonomic levels: the genus level for the overall community and the species and sub-species level for the genus *Legionella*. Ideally, all bacterial species should be identified with such a profile but the 16S rRNA genes used in our NGS approach do not provide this level of taxonomic resolution for all relevant genera. For example, for the genus *Streptococcus* only the alpha-hemolytic streptococci, including *S. pneumoniae*, can be separated from the anaerobic *S. milleri* group^23^, where as for the genus *Legionella* the resolution is sometimes better than the species level as observed for *L. pneumophila*. Other taxonomic marker genes, such as *gyrB* or *rpoB*, might offer better taxonomic resolution but await further development and application to clinical specimens^48^,^49^.

Since deep sequencing is currently not used as a diagnostic tool in hospital laboratories, further studies at larger scales are essential to assess the correlation between *Legionella* abundance and other pathogens in sputum samples, and to compare those results to the clinical and laboratory indicators such as inflammatory markers related to the presence of *Legionella.* Future studies should include a comparison of sputum with bronchoalveolar lavage (BAL) in order to assess the transfer of *Legionella* from the lung to the sputum and the correlation between *Legionella* abundance in sputum and BAL^50^. The potential of molecular sputum analyses compared to BAL and culture-based methods could be assessed with the aim to generate a fast, sensitive, and reliable diagnostic tool for *Legionella* species detection in sputum without the need of BAL. Finally, NGS profiles from sputum could help select the antibiotic mix needed for a successful treatment of pneumonia based on knowledge of the bacterial infections present.

Another aspect of potential relevance for pathology and prevention is the observation that the bacterial community occurring with high *Legionella* abundance was distinct from low or no *Legionella* presence in sputum. First, this may indicate that a certain level of *L. pneumophila* is needed to cause Legionellosis. Second, the accompanying microbiota may indicate that the microbiome of amoeba from drinking water is transferred together with the *Legionella* itself. The relevance of amoeba as a transfer vehicle of highly virulent *Legionella* may be additionally emphasized by this observation. These aspects deserve future more ample studies for insights into the adherent mechanisms of pathogenesis and potential prevention measures.

**In conclusion,** the NGS approach allowed the identification of the sputum microbiota at the genus level, and for *Legionella* genus, at the species and sub-species level. *Legionella* sub-species profiles (based on LTUs) provided patient-specific signatures. *Legionella* was never the dominant species and it is possible that in some patients coinfection with other bacterial species might have occurred. We are at the beginning of the NGS technological era. These novel techniques should certainly be considered as tools for developing new and fast molecular methods to diagnose pathogens in pneumonia patients. *Legionella* detection in sputum as well as in water samples plays a critical role in public health. Thus, identifying *Legionella* as the causative agent of infection is crucial for disease treatment and outbreak prevention^33^,^51^.

## Materials and Methods

### Ethics Statement

The methods were carried out in “accordance” with the relevant guidelines of Helsinki Committee. Written informed consent signed by each of the pneumonia patients was provided before sputum samples were taken. This study was approved by the institutional Helsinki Committee (approval no. 2013-5999).

### Sputum sampling

Sputum samples were collected from 133 pneumonia patients who were hospitalized at Poriya Hospital (Israel), between April 2013 and September 2014. All sputum samples were taken before the patients received any antibiotic treatment. All the patients were tested for influenza A (including H1N1), influenza B and respiratory syncytial virus (RSV). Only patients negative to these viruses were including in the research. All the sputum samples were stored for up to 6 hours at 4 °C and were further treated as described below in the same day of the sampling. The average age of the patients was 62.5 years; 37 patients were females and 96 males. Most of the patients were from the respiratory intensive care unit (ICU) (52%). Others were from the internal medicine department (24%), the cardiac ICU (19%), and the pediatrics department (5%).

### Culture-dependent detection of *Legionella* species

For *Legionella* culturing, 10 μl sputum samples were treated thermally (10 min, 56 °C) and then inoculated on GVPC (Glycine-Vancomycin-Polymyxin-Cycloheximide; BD, United States) *Legionella*-selective agar plates. Plates were incubated at 37 °C for 7 days. Identification of *Legionella pneumophila* was made based on morphological and Gram stain; colonies that were Gram-negative were further analyzed using a *Legionella* latex agglutination kit (Oxoid, Thermo Scientific). Colonies that were found positive were kept in Luria Broth (LB) supplemented with 30% glycerol at −80 °C.

### Culture-independent detection of *Legionella* species

For sputum DNA extraction, a protocol used for *Mycobacterium tuberculosis* detection was adapted with some modifications^52^. Briefly, 1 ml of sputum sample was mixed with MycoPrep (Becton Dickenson, USA) in a ratio of 1:1 and incubated for 17 min at room temperature. NPC-67 neutralizing buffer (Alpha Tec Systems, Inc., USA) was added to a final volume of 25 ml. Samples were centrifuged for 15 min at 3,500 g. The supernatant was discarded and the pellet was suspended in 1 ml Pellet Resuspension Buffer (Alphatec, USA). After mixing, 200 μl of the sample was used for DNA extraction using DNeasy Blood & Tissue Kit (Qiagen, Germany), according to the manufacturer’s instructions.

Genomic DNA from sputum was used as a template for amplifying *Legionella* genus-specific 16S rRNA genes according to Kahlisch *et al*.^26^. Primers specific for the genus *Legionella* were: Lgsp28R 5′-CACCGGAAATTCCACTACCCTCTC-3′ and Lgsp17F 5′-GGCCTACCAAGGCGACGATCG-3′. To verify the identification of *Legionella* from positive DNA sputum samples, PCR products were run on an agarose gel and bands from all the positive samples were excised. DNA was extracted from the bands using the QIAquick Gel Extraction Kit (Qiagen, Germany). Amplicons were sequenced by MCLAB laboratory (CA, USA). The obtained sequences were compared to those available at the EzTaxon server (http://eztaxon-e.ezbiocloud.net/)^53^ to ascertain their closest relatives. Nucleotide sequence accession numbers of the band sequences were deposited in the GenBank (KT382274-KT382277) (some of the sequences were less than 200 bp in length and thus were not deposited).

### 16S rRNA gene library preparation for microbiome analyses

Sputum DNA was amplified using primers targeting the V4 variable region of the bacterial 16SrRNA gene. Primer sequences were: CS1_515F 5′-ACACTGACGACATGGTTCTACAGTGCCAGCMGCCGCGGTAA-3′ and CS2_806R 5′-TACGGTAGCAGAGACTTGGTCTGGACTACHVGGGTWTCTAAT-3′ with an amplicon size of 291 bp^54^. These primers contained 5′ common sequence tags, in accordance to Moonsamy *et al*.^55^. The PCR amplification procedure is described in detail in Aizenberg-Gershtein *et al*.^56^.

### Illumina MiSeq sequencing

MiSeq sequencing was performed at the DNA Services (DNAS) Facility–University of Illinois, Chicago (UIC). The sequencing protocol is described in Aizenberg-Gershtein *et al*.^56^. The procedure included a second PCR amplification in 96-well plates, where each well received a separate primer pair, obtained from the Access Array Barcode Library for Illumina Sequences [10-base barcode (Fluidigm, South San Francisco, CA; Item no. 100-4876)]. Pooled, diluted libraries were sequenced using a MiSeq 600-cycle sequencing kit version 3, and analyzed with Casava1.8 (pipeline 1.8). Reads were 200 nucleotides in length (paired end, 2×200). PhiX DNA was used as a spike-in control. Barcode sequences were provided to the MiSeq server, and were automatically binned according to 10-base multiplex identifier (MID) sequences. Raw reads were recovered as FASTQ files.

### Sequence analyses of all bacteria

Bioinformatics were performed using MOTHUR v.1.33.3^57^. The MiSeq Standard Operating Procedure (SOP) followed was the one described by Kozich *et al*.^58^. Briefly, any sequences with ambiguities or homopolymers longer than 8 bases were removed from the data set. Sequences were aligned using the SILVA-compatible alignment database available within MOTHUR. Sequences were trimmed to a uniform length of 290 bp, and chimeric sequences were removed using Uchime^59^. Sequences were classified using the MOTHUR-formatted version of the RDP training set (v.9) and clustered using Furthest Neighbor algorithm, into OTUs, based on 97% sequence identity. The whole dataset was randomly subsampled to the minimum number (7,155) of sequences per sample, to avoid bias connected with uneven sequences across the samples.

The Illumina sequences can be downloaded at http://www.ncbi.nlm.nih.gov/sra. The accession for the submission is: PRJNA312879 (SRP070932).

### Microbial richness and coverage estimations

Community richness (Chao1 estimator), community diversity (coverage), and rarefaction curves were generated using the MOTHUR program (version 1.33.3). Yue and Clayton-based distance matrix, which measures community structure by incorporating both membership and abundance, was used to generate Principal Coordinates Analysis (PCoA). The significance of differences in theta index scores between the samples was assessed by analysis of molecular variance (AMOVA). AMOVA tests whether the centers of the clouds of samples representing each group (between-groups variation) are more separated than the variation among the samples of the same group (within-group variation)^60^. The groups tested were: 1. *Legionella-*positive sputum samples versus *Legionella* negative sputum samples; 2. Sputum samples for different age groups; 3. Sputum samples from different hospital departments.

### Specific sequence analyses of *Legionella* species

Illumina reads that were classified as *Legionella* spp. (n = 3,838; average read length: 292 bp) were cleared of singletons (resulting in 3,562 reads resembling 260 original sequences) and further aligned with reference sequences of all described *Legionella* species (http://www.bacterio.net, last accessed October 27, 2015) using the default settings of the ClustalW tool in MEGA (v. 6)^61^. Aligned sequences were then grouped into clusters of 99% sequence similarity using the assembly algorithm for dirty data implemented in Sequencher (v. 5.2.4), resulting in 178 clusters. For each cluster, read numbers of all individual sequences were summed and a representative sequence was picked as to serve as the “Legionella operational taxonomic unit” (LTU). A phylogenetic comparison was conducted for LTUs with more than 3 reads in total by the Neighbor Joining method under the Kimura 2-parameter model with gamma distributed rate variation among sites (shape parameter = 5) using MEGA. The phylogenetic tree was tested by bootstrapping 1000 replicates. The R package “phyloseq” (v. 1.10.0) was used for integration of abundance data and phylogeny as well as for visualization^62^.

## Additional Information

**How to cite this article**: Mizrahi, H. *et al*. Comparison of sputum microbiome of legionellosis-associated patients and other pneumonia patients: indications for polybacterial infections. *Sci. Rep.* **7**, 40114; doi: 10.1038/srep40114 (2017).

**Publisher's note:** Springer Nature remains neutral with regard to jurisdictional claims in published maps and institutional affiliations.

## Supplementary Material

Supplementary Dataset

## Figures and Tables

**Table 1:** Holm-corrected p values of differences between the richness and diversity estimates displayed in Figs 2 and S3.

Diversity indices	Observed	Chao 1	ACE	Shannon	Simpson	Inv Simpson	Fisher
**Genus level**							
*LGP/LGN*	0.082	0.11	0.096	0.3	0.94	0.22	0.069
*High-LGP/LGN*	**0.00024**	**0.0032**	**0.0028**	**0.014**	0.127	**0.037**	**8.20E-05**
*High-LGP/Low-LGP*	**0.00052**	**0.0062**	**0.0065**	**0.014**	**0.045**	**0.044**	**2.00E-04**
*Low-LGP/LGN*	0.90372	0.9947	0.9245	0.567	0.2	0.942	0.9
**Family level**							
*LGP/LGN*	0.15	0.25	0.21	0.24	0.66	0.11	0.13
*High-LGP/LGN*	**0.0026**	**0.023**	**0.018**	**0.011**	0.09	**0.01**	**0.0013**
*High-LGP/Low-LGP*	**0.0033**	**0.023**	**0.021**	**0.011**	0.064	**0.023**	**0.0018**
*Low-LGP/LGN*	0.7869	0.777	0.858	0.702	0.391	0.858	0.7893

Bold numbers indicate significant differences (p-value < 0.05).

## References

[b1] Fields B. S., Benson R. F. & Besser R. E. *Legionella* and Legionnaires’ disease: 25 years of investigation. Clin. Microbiol. Rev. 15, 506–526 (2002). 12097254 10.1128/CMR.15.3.506-526.2002 PMC118082

[b2] Phin N. . Epidemiology and clinical management of Legionnaires’ disease. Lancet Infect. Dis. 14, 1011–1021 (2014). 24970283 10.1016/S1473-3099(14)70713-3

[b3] Weissenberger C. A., Cazalet C. & Buchrieser C. *Legionella pneumophila*–a human pathogen that co-evolved with fresh water protozoa. Cell Mol. Life Sci. 64, 432–448 (2007). 17192810 10.1007/s00018-006-6391-1 PMC11138458

[b4] Diederen B. M. W. *Legionella* spp. and Legionnaires’ disease. J. Infect. 56, 1–12 (2008). 17980914 10.1016/j.jinf.2007.09.010

[b5] Daumas A. . Acute tubulointerstitial nephritis complicating Legionnaires’ disease: a case report. J. Med. Case Rep. 6, 100, doi: 10.1186/1752-1947-6-100 (2012). 22475340 PMC3359167

[b6] Cunha B. A., Burillo A. & Bouza E. Legionnaires’ disease. Lancet. 387, 376–85 (2016). 26231463 10.1016/S0140-6736(15)60078-2

[b7] Fraser D. W., Tsai T. R., Orenstein W., Parkin W. E. & Beecham H. J. Legionnaires’ disease: description of an epidemic of pneumonia. N. Engl. J. Med. 297, 1189–1197 (1977). 335244 10.1056/NEJM197712012972201

[b8] Murray P. R., Baron E. J., Jorgensen J. H., Landry M. L. & Pealler M. A. Manual of clinical microbiology 9th edn, Vol. 1. Bel. Air. M.D. (ASM Press, 2007).

[b9] Blázquez Garrido R. . Antimicrobial chemotherapy for Legionnaires disease: Levofloxacin versus Macrolides. Clin. Infect. Dis. 40, 800–806 (2005). 15736011 10.1086/428049

[b10] Plouffe F. J. . Azithromycin in the treatment of *Legionella* pneumonia requiring hospitalization. Clin. Infect. Dis. 37, 1475–1480 (2003). 14614670 10.1086/379329

[b11] European Centre for Disease Prevention and Control (ECDC). Legionnaires disease in Europe, 2010 (Stockholm: ECDC, 2012).

[b12] Moran-Gilad J. . On behalf of The ESCMID Study Group for Legionella Infections (ESGLI). Molecular epidemiology of Legionnaires’ disease in Israel. Clin. Microbiol. Infect. 20, 690–696 (2014). 24118162 10.1111/1469-0691.12425

[b13] Rodriguez-Martinez S., Sharaby Y., Brettar I., Pecellin M., Höfle M. G. & Halpern M. Spatial distribution of *Legionella pneumophila* MLVA-genotypes in a drinking water system. Water Res. 77, 119–132 (2015). 25864003 10.1016/j.watres.2015.03.010

[b14] Blanky M., Rodríguez-Martínez S., Halpern M. & Friedler E. *Legionella pneumophila*: from potable water to treated greywater; Quantification and removal during treatment. Sci. Total. Environ. 533, 557–565 (2015). 26188406 10.1016/j.scitotenv.2015.06.121

[b15] Steinert M., Hentschel U. & Hacker J. *Legionella pneumophila*: an aquatic microbe goes astray. Microbiol. Rev. 26, 149–162 (2006). 10.1111/j.1574-6976.2002.tb00607.x 12069880

[b16] Murdoch D. Diagnosis of *Legionella* infection. Medical. Microbiol. 36, 64–69 (2002). 10.1086/345529 12491204

[b17] Gadsby N. J. . Molecular diagnosis of *Legionella* infections–clinical utility of front-line screening as part of a pneumonia diagnostic algorithm J. Infect. 72, 161–170 (2016). 26632328 10.1016/j.jinf.2015.10.011

[b18] Botelho-Nevers E. . Prospective evaluation of RT-PCR on sputum versus culture, urinary antigens and serology for Legionnaire’s disease diagnosis. J. Infect. 73, 123–128 (2016). 27306488 10.1016/j.jinf.2016.04.039

[b19] Beaute J., Zucs P. & de Jong B. Legionnaires disease in Europe, 2009–2010. Euro. Surveill. 18, 20417 (2013). 23515061 10.2807/ese.18.10.20417-en

[b20] Human Microbiome Project Consortium. Structure, function and diversity of the healthy human microbiome. Nature. 486, 207–214 (2012). 22699609 10.1038/nature11234 PMC3564958

[b21] Krishna P., Jain A. & Bisen P. S. Microbiome diversity in the sputum of patients with pulmonary tuberculosis. Eur. J. Clin. Microbiol. Infect. Dis. 35, 1205–1210 (2016). 27142586 10.1007/s10096-016-2654-4

[b22] Wu J. . Sputum Microbiota Associated with New, Recurrent and Treatment Failure Tuberculosis. PLoS ONE. 8, e83445 (2013). 24349510 10.1371/journal.pone.0083445 PMC3862690

[b23] Kramer R. A. . High individuality of respiratory bacterial communities in a large cohort of adult cystic fibrosis patients under continuous antibiotic treatment. PLOS One 10, e0117436 (2015). 25671713 10.1371/journal.pone.0117436 PMC4324987

[b24] Liou Z. . Controlled evaluation of copper-silver ionization in erradicting *Legionella* from a hospital water distribution system. J. Infect. Dis. 169, 919–922 (1994). 8133111 10.1093/infdis/169.4.919

[b25] Koide M. Saito A., Kusano N. & Higa F. Detection of *Legionella* spp. in cooling tower water by the polymerase chain reaction method. Appl. Environ. Microbiol. 6, 1943–1946 (1993). 10.1128/aem.59.6.1943-1946.1993 PMC182188 8328811

[b26] Kahlisch L., Henne K., Draheim M., Brettar I. & Höfle M. G. High-resolution *in situ* genotyping of *Legionella pneumophila* populations in drinking water by multiple-locus variable-number tandem-repeat analysis using environmental DNA. Appl. Environ. Microbiol. 76, 6186–6195 (2010). 20656879 10.1128/AEM.00416-10 PMC2937494

[b27] Armstrong T. W. & Haas C. N. A quantitative microbial risk assessment model for Legionnaires’ disease: animal model selection and dose-response modelling. Risk Anal. 27, 1581–1596 (2007). 18093054 10.1111/j.1539-6924.2007.00990.x

[b28] Mandell D. L., Bennett J. E. & Dolin R. Principles and practice of infectious disease 7th edn, Vol. 2. New York (Churchill Livingstone, 2010).

[b29] Whiley H., Keegan A., Fallowfield H. & Ross K. Uncertainties associated with assessing the public health risk from Legionella. Frontiers Microbiol. 5, Article 501 (2014). 10.3389/fmicb.2014.00501 PMC4174118 25309526

[b30] Delafont V., Brouke A., Bouchon D., Moulin L. & Héchard Y. Microbiome of free-living amoeba from drinking water. Water Res. 47, 6958–6965 (2013). 24200009 10.1016/j.watres.2013.07.047

[b31] Thomas V., Herrera-Rimann K., Blanc D. S. & Greub G. Biodiversity of amoeba and amoeba-resisting bacteria in a hospital water network. Appl. Environ. Microbiol. 72, 2429–2438 (2006). 10.1128/AEM.72.4.2428-2438.2006 PMC1449017 16597941

[b32] Thomas V., McDonnell G., Denyer S. P. & Maillard J.-Y.. Free-living amoebae and their intracellular pathogenic microorganisms: risks for water quality. FEMS Microbiol. Rev. 34, 231–259 (2010). 19744244 10.1111/j.1574-6976.2009.00190.x

[b33] Cho M. C. . Comparison of sputum and nasopharyngeal swab specimens for molecular diagnosis of *Mycoplasma pneumoniae, Chlamydophila pneumoniae*, and *Legionella pneumophila*. Ann. Lab. Med. 32, 133–138 (2012). 22389880 10.3343/alm.2012.32.2.133 PMC3289778

[b34] Hauser M. P. . Microbiota present in cystic fibrosis lungs as revealed by whole genome sequencing. Plos ONE 9, 1036–1040 (2014). 10.1371/journal.pone.0090934 PMC3944733 24599149

[b35] Tan M. J., Tan S. J. & File T. M. Legionnaires disease with bacteremic coinfection. Clin. Infect. Dis. 35, 533–539 (2002). 12173126 10.1086/341771

[b36] Takayanagi N. . Polymicrobial infections in patients with *Legionella pneumonia*. (in Japanese). Nihon Kokyuki Gakkai Zasshi 42, 62–67 (2004). 14768366

[b37] Lerolle N., Zahar J. R., Duboc V., Tissier F. & Rabbat A. Pneumonia involving *Legionella pneumophila* and *Listeria monocytogenes* in an immunocompromised patient: an unusual coinfection. Respiration. 69, 359–361 (2002). 12169753 10.1159/000063263

[b38] Musallam N. . *Legionella pneumophila* and *Pneumocystis jirovecii* coinfection in an infant treated with adrenocorticotropic hormone for infantile spasm: case report and literature review. J. Child Neurol. 29, 240–242 (2014). 24309244 10.1177/0883073813511148

[b39] Rizzo C., Caporali M. G. & Rota M. C. Pandemic Influenza and Pneumonia Due to *Legionella pneumophila*: A Frequently Underestimated Coinfection. Clin. Infect. Dis. 51, 115 (2010). 10.1086/653444 20518675

[b40] Oggioni C. . Legionnaires’ disease contracted from patient workplace: First report of a severe case of coinfection with varicella-zoster virus. Am. J. Infect. Control, doi: 10.1016/j.ajic.2016.03.057 (2016). 27311515

[b41] Charlson E. S. . Topographical continuity of bacterial populations in the healthy human respiratory tract. Am. J. Respir. Crit. Care Med. 184, 957–63 (2011). 21680950 10.1164/rccm.201104-0655OC PMC3208663

[b42] Morris A. . Comparison of the respiratory microbiome in healthy nonsmokers and smokers. Am. J. Respir. Crit. Care Med. 187, 1067–1075 (2013). 23491408 10.1164/rccm.201210-1913OC PMC3734620

[b43] Hung Y. J. . The role of the lung microbiome in health and disease. Am. J. Respir. Crit. Care Med. 187, 1382–1387 (2013). 23614695 10.1164/rccm.201303-0488WS PMC5155250

[b44] Segal L. N. . Enrichment of the lung microbiome with oral taxa is associated with lung inflammation of a Th17 phenotype. Nat. Microbiol. 1, 1603, doi: 10.1038/nmicrobiol.2016.31 (2016). PMC5010013 27572644

[b45] Coscollá M., Fernández C., Colomina J., Sánchez-Busó L. & González-Candelas F. Mixed infection by *Legionella pneumophila* in outbreak patients. Int. J. Med. Microbiol. 304, 307–313 (2014). 24309206 10.1016/j.ijmm.2013.11.002

[b46] Peters B. M., Jabra-Rizk M. A., O’May G. A., Costerton J. W. & Shirtliff M. E. Polymicrobial interactions: Impact on pathogenesis and human disease. Clin. Microbiol. Rev. 25, 193–213 (2012). 22232376 10.1128/CMR.00013-11 PMC3255964

[b47] Short F. L., Murdoch S. L. & Ryan R. P. Polybacterial human disease: the ills of social networking. Trends Microbiol. 22, 508–516 (2014). 24938173 10.1016/j.tim.2014.05.007 PMC4158425

[b48] Adekambi T., Drancourt M. & Raoult D. The *rpoB* gene as a tool for clinical microbiologists. Trends Microbiol. 17, 37–45 (2009). 19081723 10.1016/j.tim.2008.09.008

[b49] Das S., Dash H. R., Mangwani N., Chakraborty J. & Kumari S. Understanding molecular identification and polyphasic taxonomic approaches for genetic relatedness and phylogenetic relationships of microorganisms. J. Microbiol. Meth. 103, 80–100 (2014). 10.1016/j.mimet.2014.05.013 24886836

[b50] Jahn K. . Molecular diagnostics for bacterial infections in bronchoalveolar lavage–a case-control, pilot study. Swiss Med. Wkly. 145, w14193 (2015). 26715104 10.4414/smw.2015.14193

[b51] White P. S. . Epidemiological investigation of a Legionnaires’ disease outbreak in Christchurch, New Zealand: the value of spatial methods for practical public health. Epidemiol. Infect. 141, 789–799 (2013). 22697112 10.1017/S0950268812000994 PMC11616728

[b52] Diacon A. H. . Time to detection of the growth of *Mycobacterium tuberculosis* in MGIT 960 for determining the early bactericidal activity of antituberculosis agents. Eur. J. Clin. Microbiol. Infect. Dis. 29, 1561–1565 (2010). 20820832 10.1007/s10096-010-1043-7

[b53] Kim O. S. . Introducing EzTaxon-e: a prokaryotic 16S rRNA gene sequence database with phylotypes that represent uncultured species. Int. J. Syst. Evol. Microbiol. 62, 716–721 (2012). 22140171 10.1099/ijs.0.038075-0

[b54] Caporaso J. G. . Ultra-high-throughput microbial community analysis on the Illumina HiSeq and MiSeq platforms. ISME J. 6, 1621–1624 (2012). 22402401 10.1038/ismej.2012.8 PMC3400413

[b55] Moonsamy P. V. . High throughput HLA genotyping using 454 sequencing and the Fluidigm Access Array™ system for simplified amplicon library preparation. Tissue Antigens. 81, 141–149 (2013). 23398507 10.1111/tan.12071

[b56] Aizenberg-Gershtein Y. . Pyridine-type alkaloid composition affects bacterial community composition of floral nectar. Sci. Rep. 5, 11536 (2015). 26122961 10.1038/srep11536 PMC4650603

[b57] Schloss P. D. . Introducing mothur: open-source platform-independent community-supported software for describing and comparing microbial communities. Appl. Environ. Microbiol. 75, 7537–7541 (2009). 19801464 10.1128/AEM.01541-09 PMC2786419

[b58] Kozich J. J., Westcott S. L., Baxter N. T., Highlander S. K. & Schloss P. D. Development of a dual-index sequencing strategy and curation pipeline for analyzing amplicon sequence data on the MiSeq Illumina sequencing platform. Appl. Environ. Microbiol. 79, 5112–5120 (2013). 23793624 10.1128/AEM.01043-13 PMC3753973

[b59] Edgar R. C., Haas B. J., Clemente J. C., Quince C. & Knight R. UCHIME improves sensitivity and speed of chimera detection. Bioinformatics. 27, 2194–2200 (2011). 21700674 10.1093/bioinformatics/btr381 PMC3150044

[b60] Excoffier L., Smouse P. E. & Quattro J. M. Analysis of molecular variance inferred from metric distances among mtDNA haplotypes: application to human mitochondrial DNA restriction data. Genetics. 131, 479–491 (1992). 1644282 10.1093/genetics/131.2.479 PMC1205020

[b61] Tamura K., Stecher G., Peterson D., Filipski A. & Kumar S. MEGA6: Molecular evolutionary genetics analysis Version 6.0. Mol. Biol. Evol. 30, 2725–2729 (2013). 24132122 10.1093/molbev/mst197 PMC3840312

[b62] McMurdiem P. J. & Holmes S. phyloseq: An R Package for reproducible interactive analysis and graphics of microbiome census data. PLoS ONE 8, e61217 (2013). 23630581 10.1371/journal.pone.0061217 PMC3632530

