# Supplementary material for: Comparison of sputum microbiome of legionellosis-associated patients and other pneumonia patients: indications for polybacterial infections
Source: Sci Rep. 2017 Jan 6;7:40114. doi: 10.1038/srep40114 (PMC5216348; doi:10.1038/srep40114)
Supplement: Supplementary Dataset [file srep40114-s1.pdf]

Comparison of sputum microbiome of legionellosis-associated patients and other pneumonia patients: indications for polybacterial infections

Hila Mizrahi, Avi Peretz, René Lesnik, Yana Aizenberg-Gershtein, Sara Rodríguez-Martínez, Yehonatan Sharaby, Nina Pastukh, Ingrid Brettar, Manfred G. Höfle, Malka Halpern

Supplementary Figures

Fig S1. Rarefaction curves indicating the observed number of operational taxonomic units (OTUs) at a phylogenetic distance of 3% sequence similarity in the sputum samples.

Fig S2. Average OTU abundances at the phyla level. A. *Legionella*-positive (LGP) *vs*. *Legionella*-negative (LGN) samples. B. Samples with high (High-LGP) and low (Low-LGP) *Legionella* abundance *vs*. LGN.

Fig S3. Richness and diversity estimates on the genus (above) and family (below) level. For p values see Table 1.

Supplementary Tables

Table S1. Data of *Legionella* positive and negative patients. Sample 2PS2 was positive both by culture and by PCR method.

Table S2. Richness (Chao1) and microbial coverage of the sputum samples (subsampled OTU's level).

Table S3. Bacterial community composition (relative abundances as %) in *Legionella* positive (LGP) sputum samples (n = 8).

Table S4. Bacterial community composition (relative abundances as %) in *Legionella* negative (LGN) sputum samples (n = 13).

Table S5. Summary of the adjusted number of reads for the most abundant *Legionella* spp. clusters in LGP sputum samples.

Table S6. Results from the Illumina bacterial communities' analyses compared to the results from the hospital laboratory culture.

Figures

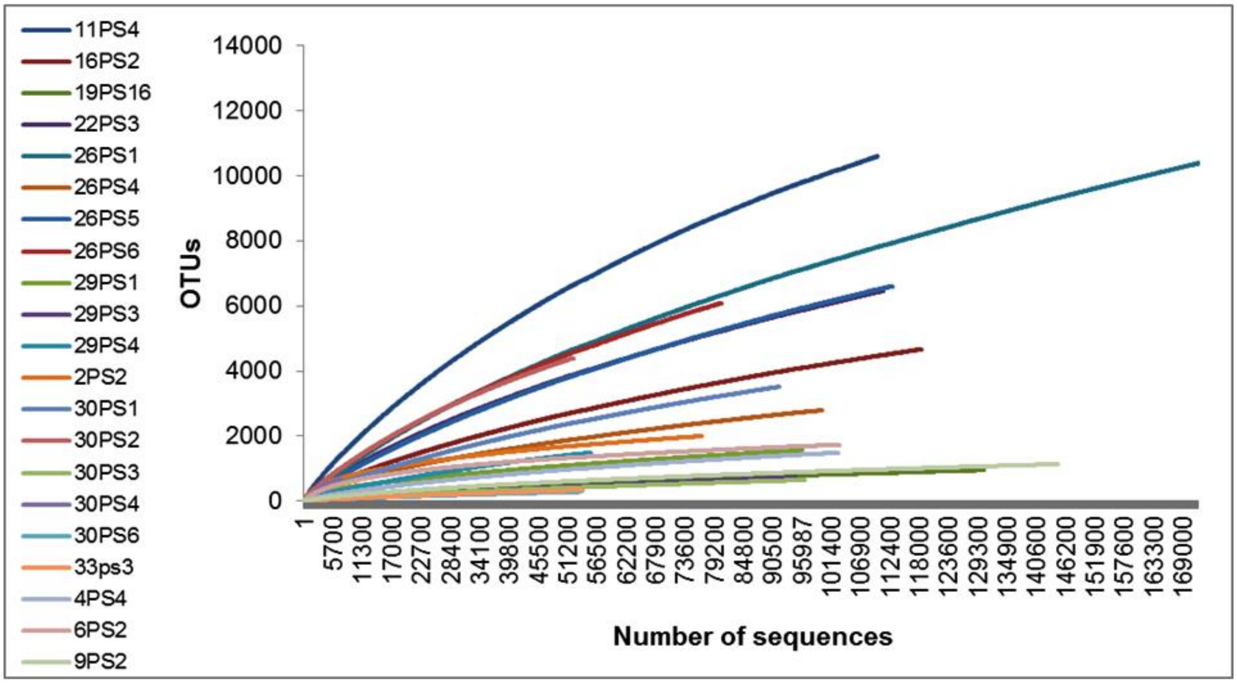

Fig S1. Rarefaction curves indicating the observed number of operational taxonomic units (OTUs) at a phylogenetic distance of 3% sequence similarity in the sputum samples.

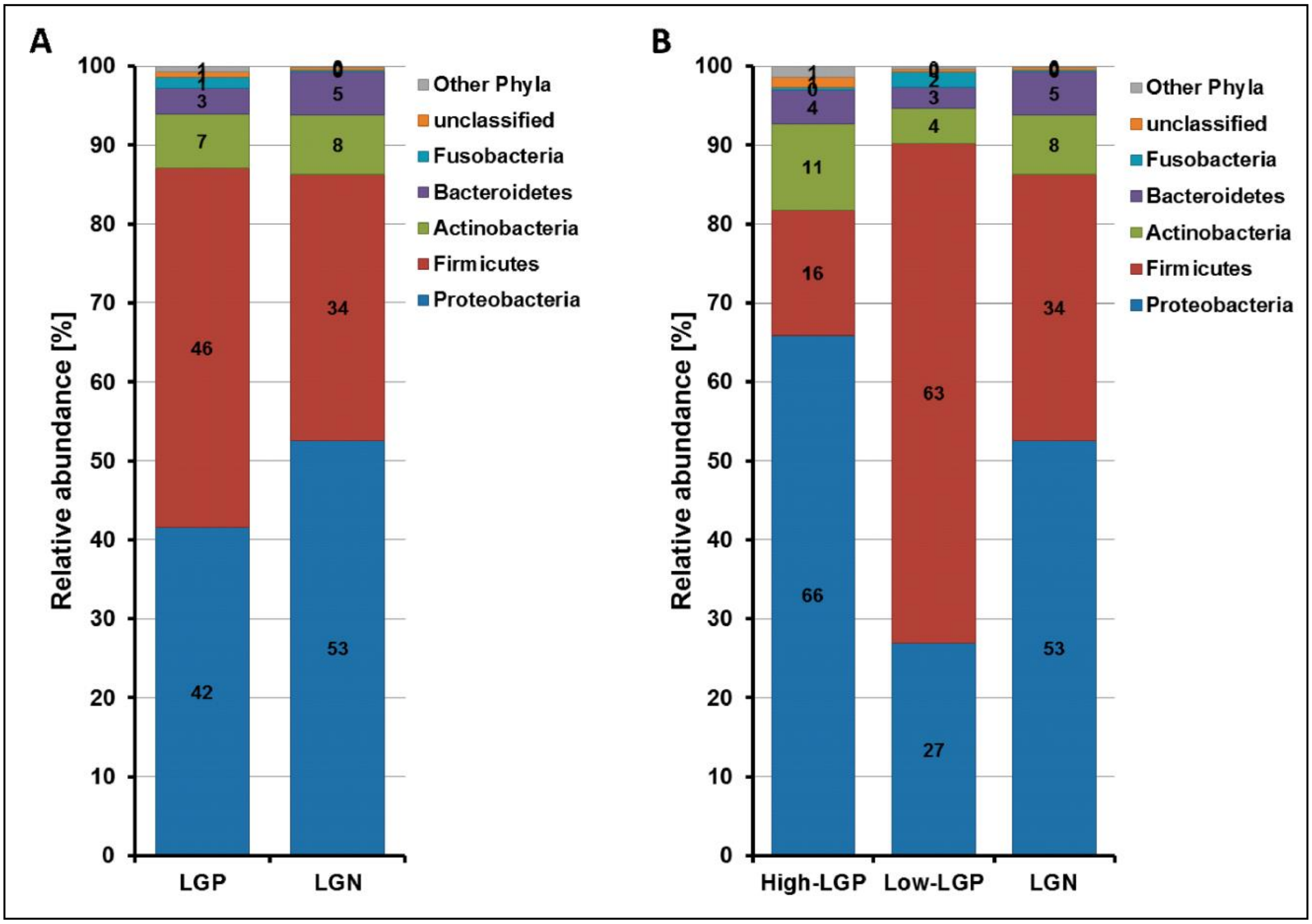

Fig S2. Average OTU abundances at the phyla level. A. *Legionella*-positive (LGP) *vs*. *Legionella*-negative (LGN) samples. B. Samples with high (High-LGP) and low (Low-LGP) *Legionella* abundance *vs*. LGN.

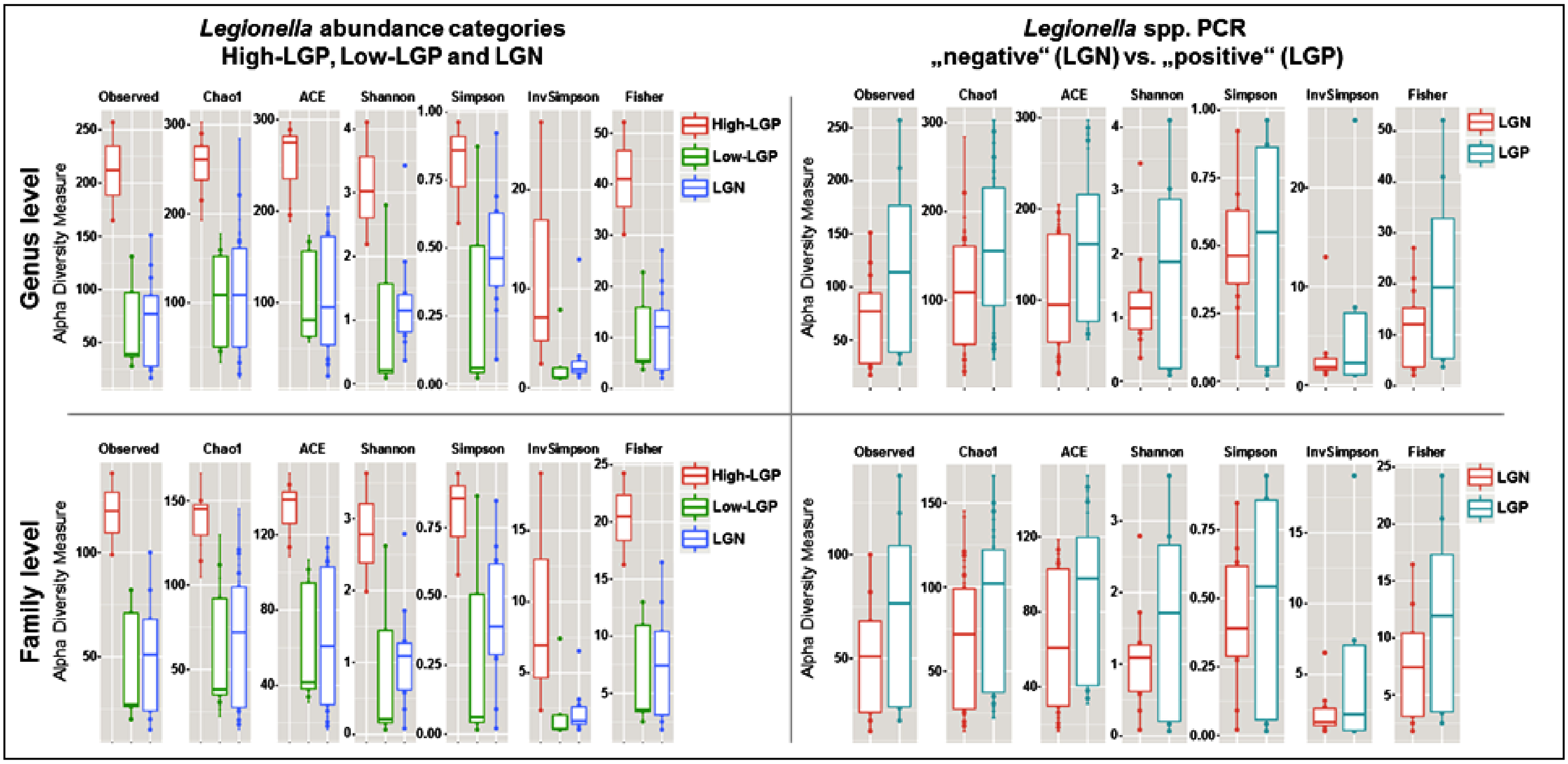

Fig S3. Richness and diversity estimates on the genus (above) and family (below) level. For p values see Table 1.

Tables

Table S1. Data of *Legionella* positive and negative patients. Sample 2PS2 was positive both by culture and by PCR method. ICU, intensive care unit; NA, data not available.

**Sample name**	**Age**	**Gender**	**Department**	**Sampling date**
***Legionella* positive patients**				
2PS2	81	Female	Respiratory ICU	19.03.2013
6PS2	58	Male	Respiratory ICU	12.08.2013
16PS2	65	Female	Geriatric department	13.10.2013
16PS4	0	Female	Pediatrics department	13.10.2013
26PS1	56	Male	Cardiac ICU	27.04.2014
26PS4	52	Male	Respiratory ICU	27.04.2014
26PS5	45	Male	Cardiac ICU	27.04.2014
26PS6	56	Female	Respiratory ICU	27.04.2014
33PS3	83	Male	Respiratory ICU	01.09.2014
***Legionella* negative patients**				
11PS4	50	NA	Cardiac ICU	11.09.2013
19PS6	64	NA	Respiratory ICU	16.01.2014
22PS3	90	NA	Cardiac ICU	26.0.2014
29PS3	70	NA	Respiratory ICU	29.06.2014
29PS4	46	NA	Respiratory ICU	29.06.2014
30PS1	55	NA	Internal medicine	06.07.2014
30PS2	64	NA	Internal medicine	06.07.2014
30PS3	79	NA	Internal medicine	06.07.2014
30PS4	44	NA	Respiratory ICU	06.07.2014
30PS6	77	NA	Respiratory ICU	06.07.2014
4PS4	75	NA	internal medicine	17.07.2013
9PS2	84	NA	Internal medicine	26.08.2013

Table S2. Richness (Chao1) and microbial coverage of the sputum samples (subsampled OTU's level). Chao1 is an estimator of the expected taxonomic richness (see details in the Methods section). LGP, *Legionella* positive sample; LGN, *Legionella* negative sample.

**Group**	**Sample name**	**Chao 1**	**Coverage**
LGP	16PS2	2079	0.95
LGP	26PS1	3342	0.96
LGP	26PS4	1279	0.96
LGP	26PS5	3995	0.93
LGP	26PS6	4892	0.89
LGP	2PS2	1202	0.95
LGP	33PS3	346	0.99
LGP	6PS2	948	0.96
LGN	29PS1	733	0.97
LGN	29PS3	416	0.99
LGN	29PS4	1506	0.96
LGN	30PS1	2310	0.95
LGN	30PS2	4757	0.88
LGN	30PS3	338	0.99
LGN	30PS4	1300	0.94
LGN	30PS6	253	0.99
LGN	4PS4	836	0.98
LGN	9PS2	343	0.99
LGN	11PS4	6535	0.87
LGN	19PS16	334	0.99
LGN	22PS3	3664	0.92

Table S3. Bacterial community composition (relative abundances as %) in *Legionella* positive (LGP) sputum samples (n = 8). The table presents OTUs at the genus level, whose prevalence in the sequence reads were at least 0.5 % in one of the samples. Samples are sorted with decreasing abundance of *Legionella* (first row). Taxa (genera) are sorted with decreasing average abundance across all samples. The last column refers to the rank abundance curves (A, B, C) in Fig 5. Note that the sums of unclassified genera are relatively high in samples 2PS2 and 6PS2. Abundance levels are bold if equal or above 2.0% and marked with "b" if below 0.05%, a minus ("-") indicates that the specific sample contained no reads for that genus.

	**High-LGP (n = 3)**			**Low-LGP (n = 5)**					
**Genus**	**2PS2***	**6PS2**	**26PS4**	**26PS6**	**26PS1**	**26PS5**	**16PS2**	**33PS3****	**Abundance category**
Legionella abundance	**2.88**	0.82	0.56	0.11	0.04	0.03	0.02	0.004	B
Streptococcus	**2.2**	0.6	**17.9**	**70.9**	**97.7**	**97.1**	**29.2**	-	A
unclassified genera	**27.1**	**72.9**	**8.5**	**5.8**	0.8	1.0	**10.5**	0.5	A
*Acinetobacter****	**13.4**	1.8	**31.3**	1.3	0.1	0.2	0.2	**98.9**	A
*Stenotrophomonas****	0.2	0.1	1.9	0.5	0.2	b	b	-	A
*Escherichia/Shigella****	0.2	1.3	0.5	b	b	-	b	-	A
Haemophilus	b	b	**7.0**	b	0.1	b	b	-	A
Proteus	b	b	b	-	b	b	**2.1**	-	A
Corynebacterium	**2.9**	1.2	0.7	0.1	0.3	b	**6.7**	-	A
Prevotella	0.9	b	1.5	1.8	b	-	**2.9**	-	A
Staphylococcus	1.1	**2.2**	**2.0**	0.5	b	b	0.1	-	A
Paracoccus	**7.4**	1.2	0.2	0.1	b	b	b	b	B
Rothia	0.1	b	0.2	**7.0**	0.1	b	**3.6**	-	B
Providencia	-	b	0.2	-	-	-	**3.3**	-	B
Veillonella	1.4	0.1	**2.5**	**4.4**	b	b	0.7	-	B
Neisseria	0.1	0.1	1.0	b	0.2	b	**15.2**	-	B
Gemella	0.1	b	1.1	1.0	0.1	0.9	0.8	-	B
Fusobacterium	0.4	b	0.4	0.1	b	b	**8.9**	-	B
Cellulomonas	0.1	b	-	-	-	-	b	-	B
Lactobacillus	**2.2**	0.1	b	0.5	-	-	b	-	B
Micrococcus	**4.7**	**2.5**	0.4	0.2	b	b	0.1	-	B
*Pseudomonas****	1.8	0.4	1.9	0.3	0.1	b	b	-	B
Delftia	0.1	0.4	b	-	-	-	b	-	B
Enterococcus	b	b	b	-	-	-	-	0.2	B
Porphyromonas	0.1	b	1.1	0.2	-	b	**4.6**	-	B
Actinomyces	0.2	0.1	0.9	1.4	b	b	0.4	-	B
Naxibacter	0.5	0.2	0.2	-	-	-	-	-	B
*Legionella****	**2.9**	0.8	0.6	0.1	b	b	b	b	B
*Lactococcus****	0.4	0.5	0.2	b	b	b	0.1	-	B
Dietzia	**2.6**	0.7	0.2	0.1	b	b	b	-	B
*Sphingomonas****	0.4	0.3	0.5	b	b	b	0.1	b	B
Vibrio	b	0.3	**3.0**	b	b	-	b	-	B
Granulicatella	0.1	b	0.6	0.6	b	0.1	0.2	-	B
Rubellimicrobium	0.2	b	-	-	-	-	-	-	B
Helicobacter	-	-	**2.5**	-	-	b	-	b	B
Flavobacterium	0.6	0.4	0.9	b	-	-	0.1	-	B

Luteimonas	1.6	0.1	-	b	b	b	b	-	B
Megasphaera	0.1	-	0.3	0.2	-	b	b	-	C
Nocardioides	0.2	b	b	b	-	-	b	-	C
Roseomonas	b	b	b	-	-	-	-	-	C
Peptostreptococcus	0.1	b	0.1	b	-	-	1.7	-	C
Rhodocytophaga	-	-	-	-	-	-	-	-	C
Exiguobacterium	0.2	0.2	b	b	-	b	b	-	C
Brachybacterium	0.8	0.2	0.6	b	b	-	-	-	C
*Brevundimonas****	0.7	0.2	0.2	b	b	b	b	-	C
Chryseobacterium	0.1	0.2	0.1	0.1	b	-	b	-	C
Atopobium	0.1	-	0.1	0.1	b	-	0.1	-	C
Aeromonas	0.1	0.4	0.7	b	-	b	b	-	C
Campylobacter	b	-	0.1	0.1	b	-	0.6	-	C
Treponema	b	b	0.4	0.1	b	b	0.6	-	C
*Curvibacter****	1.4	b	-	-	-	-	b	-	C
Capnocytophaga	b	b	0.1	b	-	b	1.3	-	C
Bifidobacterium	b	b	0.1	b	-	-	b	-	C
Dolosigranulum	b	0.2	0.2	0.1	b	b	b	-	C
Hymenobacter	0.1	-	0.1	-	-	b	b	-	C
Gemmatimonas	0.1	1.0	b	b	-	-	b	-	C
Skermanella	0.1	b	-	-	-	-	-	-	C
Chondromyces	b	-	-	-	-	-	-	-	C
Blastococcus	0.4	b	b	b	-	-	b	-	C
Belnapia	-	-	-	-	-	-	-	-	C
Leptotrichia	b	b	0.2	b	b	-	0.2	-	C
Phenylobacterium	1.0	b	-	-	-	-	b	-	C
Propionibacterium	0.2	0.5	b	b	b	b	b	-	C
Kocuria	0.1	0.1	b	0.7	-	-	b	-	C
SR1, incertae sedis	b	b	b	-	-	-	0.9	-	C
*Sphingobacterium****	0.8	b	b	b	-	b	-	-	C
*Novosphingobium****	0.9	b	b	-	-	-	-	b	C
Clostridium, sensu stricto	0.1	0.1	0.7	b	b	b	-	b	C
Catonella	-	-	-	b	-	-	0.7	-	C
Hydrogenophaga	b	0.7	-	-	-	-	b	-	C
Oribacterium	b	-	b	b	-	-	0.5	-	C
*Bradyrhizobium****	0.7	0.1	b	-	-	-	b	b	C
*Methylobacterium****	0.6	b	b	-	-	-	b	-	C
**Total**	**84.7**	**92.7**	**93.9**	**98.6**	**99.7**	**99.6**	**96.6**	**99.6**	

*Culture positive sample

** Number of *Legionella* reads too low for detailed analysis

*** Genus occurred in microbiome of amoebae in drinking water distribution system (Delafont et al. 2013)

Table S4. Bacterial community composition (relative abundances as %) in *Legionella* negative (LGN) sputum samples (n = 13). The Table presents OTUs at the genus level, whose prevalence in the sequence reads were at least 0.5% in one of the samples. Samples are sorted alphabetically. **Taxa** (genera) are sorted with decreasing average abundance across all samples. The last column (Abundance category- A, B, C), refers to the rank abundance curves in Fig. 5. Note that the sums of unclassified genera are relatively high in samples 30PS6, 4PS4 and 9PS2. Abundance levels are bold if equal or above 2.0% and marked with "b" if below 0.05%, a minus ("-") indicates that the specific sample contained no reads for that genus.

	**LGN (not detected) (n = 13)**													
**Genus**	**11PS4**	**19PS16**	**22PS3**	**29PS1**	**29PS3**	**29PS4**	**30PS1**	**30PS2**	**30PS3**	**30PS4**	**30PS6**	**4PS4**	**9PS2**	**Abundance category**
Streptococcus	**80.1**	b	**85.7**	**2.5**	0.3	**19.1**	**21.8**	**82.8**	b	**72.7**	b	1.6	0.2	A
unclassified genera (summarized)	**3.0**	1.2	**2.4**	**32.0**	**3.8**	**9.4**	**7.7**	**8.0**	**2.4**	**4.1**	**26.1**	**71.0**	**54.1**	A
Acinetobacter	0.4	0.7	0.3	**3.7**	-	b	b	b	0.5	-	b	1.5	**41.5**	A
Stenotrophomonas	b	**95.7**	b	1.5	b	-	0.1	b	**21.0**	-	b	**7.4**	b	A
Escherichia/Shigella	b	b	0.2	1.9	**95.2**	-	-	-	-	0.1	b	b	b	A
Haemophilus	0.2	b	b	1.6	-	**71.0**	0.1	0.1	b	b	b	b	b	A
Proteus	b	-	b	0.1	b	b	0.1	b	**2.2**	b	**73.0**	0.3	-	A
Corynebacterium	0.1	0.1	**3.3**	0.3	b	-	b	0.1	**55.6**	0.3	-	**2.9**	b	A
Prevotella	0.4	-	0.1	-	0.1	b	**50.6**	b	-	**7.6**	b	b	0.2	A
Staphylococcus	**2.9**	0.1	**3.7**	0.3	b	b	1.2	**4.9**	**2.0**	-	b	0.2	1.6	A
Paracoccus	b	b	b	**10.3**	-	-	-	-	-	-	-	0.1	b	B
Rothia	**4.0**	b	b	b	0.1	b	0.8	0.1	-	**2.1**	b	b	b	B
Providencia	-	-	-	-	-	-	-	-	**13.3**	-	-	0.3	-	B
Veillonella	0.2	-	b	b	0.1	b	**3.2**	b	b	**4.3**	-	b	b	B
Neisseria	b	b	0.1	0.1	b	-	b	-	-	0.1	b	-	0.1	B
Gemella	**3.4**	b	b	0.1	b	0.2	0.1	0.6	-	**3.9**	b	b	b	B
Fusobacterium	0.3	b	0.1	b	b	b	0.3	b	b	1.3	b	b	b	B
Cellulomonas	b	b	-	**10.5**	-	-	-	-	-	-	-	b	-	B
Lactobacillus	0.3	0.1	0.1	0.1	0.1	0.1	**3.5**	1.8	-	-	-	0.3	b	B
Micrococcus	0.2	0.1	0.1	0.4	-	-	0.1	-	b	-	b	b	b	B
Pseudomonas	0.1	0.1	0.1	1.2	b	b	0.1	b	b	-	0.2	1.0	0.6	B
Delftia	-	-	-	-	-	b	-	-	1.9	-	-	**4.8**	b	B
Enterococcus	b	b	-	1.3	-	-	-	-	-	-	-	**5.3**	-	B
Porphyromonas	0.1	b	0.2	b	-	b	0.1	-	-	0.3	-	b	b	B

Actinomyces	0.5	b	b	b	0.1	-	**2.5**	b	-	0.6	-	b	b	B
Naxibacter	-	0.1	b	**5.0**	-	-	-	-	-	-	-	b	-	B
Legionella	-	b	-	-	b	-	b	-	-	-	-	b	b	B
Lactococcus	0.4	0.1	b	1.3	-	-	b	1.0	-	-	-	b	b	B
Dietzia	0.1	b	0.1	0.2	-	-	b	-	-	-	-	b	b	B
Sphingomonas	b	b	0.1	**2.3**	-	-	b	-	-	-	-	0.1	0.1	B
Vibrio	b	b	b	-	-	-	b	-	b	-	-	0.1	b	B
Granulicatella	1.1	b	b	-	b	b	0.1	-	-	0.3	-	-	b	B
Rubellimicrobium	b	0.1	-	**2.6**	-	-	-	-	-	-	b	b	b	B
Helicobacter	-	-	b	-	b	-	-	-	-	-	-	-	-	B
Flavobacterium	0.1	-	b	-	-	-	-	-	-	-	-	0.2	0.1	B
Luteimonas	b	-	b	0.4	-	-	-	-	-	-	-	b	-	B
Megasphaera	b	-	-	-	b	-	1.2	-	-	0.3	-	-	b	C
Nocardioides	-	b	b	1.8	-	-	-	-	-	-	b	-	b	C
Roseomonas	-	b	b	**2.0**	-	-	-	-	-	-	-	-	-	C
Peptostreptococcus	-	-	-	b	b	-	0.1	-	-	-	-	-	b	C
Rhodocytophaga	-	-	-	**2.0**	-	-	b	-	-	-	-	b	-	C
Exiguobacterium	b	b	b	1.2	-	-	0.1	-	b	-	b	b	-	C
Brachybacterium	b	b	b	b	-	-	-	-	-	-	-	-	b	C
Brevundimonas	b	b	b	0.2	-	-	-	-	b	-	-	0.3	b	C
Chryseobacterium	0.1	b	b	-	-	-	-	-	-	-	-	1.0	b	C
Atopobium	b	-	-	-	b	-	1.1	b	-	0.1	-	-	b	C
Aeromonas	b	b	b	0.1	-	-	b	-	b	b	-	0.1	0.1	C
Campylobacter	b	-	b	b	b	b	0.2	b	-	0.4	b	-	b	C
Treponema	b	-	0.1	-	-	b	0.1	-	-	-	-	b	-	C
Curvibacter	b	-	b	-	-	-	-	-	b	-	-	b	-	C
Capnocytophaga	b	b	b	-	-	-	-	-	-	b	-	b	b	C
Bifidobacterium	b	b	0.1	-	b	-	1.1	b	-	-	b	-	b	C
Dolosigranulum	b	b	0.8	-	-	-	-	-	-	-	-	b	-	C
Hymenobacter	b	b	b	1.1	-	-	-	-	-	-	-	b	-	C
Gemmatimonas	b	b	b	0.1	-	-	b	-	-	-	-	-	-	C
Skermanella	-	b	-	1.1	-	-	-	-	-	-	-	-	-	C
Chondromyces	-	-	-	1.1	-	-	-	-	-	-	-	-	-	C
Blastococcus	b	b	b	0.6	-	-	b	-	-	-	-	b	-	C

Belnapia	-	-	-	1.1	-	-	-	-	-	-	-	-	-	C
Leptotrichia	b	-	-	b	b	b	0.6	-	-	b	-	b	-	C
Phenylobacterium	b	b	-	-	-	-	-	-	-	-	-	b	b	C
Propionibacterium	0.1	b	b	0.1	b	-	b	-	-	-	-	b	-	C
Kocuria	0.1	b	b	b	-	-	-	-	-	-	-	b	b	C
SR1, incertae sedis	-	b	-	b	-	-	-	-	-	-	-	-	b	C
Sphingobacterium	b	b	b	b	-	b	b	-	-	-	b	-	-	C
Novosphingobium	-	b	b	b	-	-	-	-	-	-	-	b	b	C
Clostridium, sensu stricto	b	b	b	b	b	b	b	-	b	b	b	b	b	C
Catonella	b	-	b	-	b	-	-	-	-	0.1	-	-	-	C
Hydrogenophaga	b	-	-	-	-	-	-	-	-	-	-	b	b	C
Oribacterium	b	-	-	-	-	b	0.1	-	-	b	-	-	b	C
Bradyrhizobium	-	-	-	b	-	-	-	-	-	-	-	-	-	C
Methylobacterium	b	b	b	b	-	-	-	-	-	-	-	b	b	C
**Total**	**98.5**	**98.8**	**98.1**	**92.5**	**99.9**	**99.8**	**97.0**	**99.4**	**99.0**	**98.7**	**99.5**	**98.8**	**98.8**	

Table S5. Summary of the adjusted number of reads for the most abundant *Legionella* spp. clusters in LGP sputum samples. Only clusters that represent more than 10 *Legionella* reads in at least one of the LGP samples are listed.

**Sample**	**2PS2**	**6PS2**	**26PS4**	**26PS6**	**26PS1**	**26PS5**	**16PS2**
Cluster *L. pneumophila* Lens	1318	164	78	54	12	14	2
Cluster *L. pneumophila* Philadelphia	560	271	289	24	10	0	0
OTU 15	0	19	0	0	0	0	0
OTU 19	0	13	0	0	0	0	0
OTU 24	12	0	0	0	0	0	0
Sum of other *Legionella* OTU reads	144	36	25	0	2	0	9

Table S6. Results from the Illumina bacterial communities' analyses compared to the results from the hospital laboratory culture. *No pathogens were cultured.

**Sample**	**Dominant bacteria in sputum (%)**	**More results from sputum (%)**	**Hospital Lab sputum culture**
**Sputum from positive *Legionella* patients**			
2PS2	*Acinetobacter* (13.4)	*Legionella* (2.9)	*Legionella*
6PS2	*Micrococcus* (2.5)	*Legionella* (0.82)	-*
26PS4	*Acinetobacter* (31.3)	*Legionella* (0.56)	-*
26PS5	*Streptococcus* (97.1)	*Legionella* (0.03)	-*
33PS3	*Acinetobacter* (98.9)	*Legionella* (0.004)	*Escherichia*
**Sputum from negative *Legionella* patients**			
9PS2	*Acinetobacter* (41.5)		-*
11PS4	*Streptococcus* (80.1)		*Candida*
29PS1	*Cellulomonas* (10.4)<br>*Paracoccus* (10.2)	*Pseudomonas* (1.0)	*Pseudomonas*
29PS3	*Escherichia* (95.2)		*Escherichia*
29PS4	*Haemophilus* (71.0)	*Streptococcus* (19.1)	*Streptococcus*
30PS2	*Streptococcus* (82.8)	*Proteus* (0.01)	*Proteus*
30PS4	*Streptococcus* (72.0)	*Escherichia* (0.1)	*Escherichia*
30PS6	*Proteus* (73.0)	*Acinetobacter* (0.2)	*Acinetobacter*
